# Area of ​​residence modifies the association between sleep disorders and health-related quality of life in older adults: a cross-sectional analysis of two cohort studies in Mexico

**DOI:** 10.1186/s12889-026-27527-6

**Published:** 2026-05-13

**Authors:** Betty Manrique-Espinoza, Aarón Salinas-Rodríguez, Selene Guerrero-Zúñiga, Jessica Velasco-García, Karla Moreno-Tamayo, Sergio Sánchez-García

**Affiliations:** 1 Centro de Investigación en Evaluación y Encuestas, Instituto Nacional de Salud Pública, Av. Universidad 655, Colonia Santa María Ahuacatitlán, Cuernavaca, Morelos CP. 62100 México; 2 Clínica de Sueño, Instituto Nacional de Enfermedades Respiratorias Ismael Cosío Villegas, Ciudad de México, México; 3 Unidad de Investigación Epidemiológica y en Servicios de Salud, Área Envejecimiento, Centro Médico Nacional Siglo XXI, Instituto Mexicano del Seguro Social, Av. Cuauhtémoc 330, Edificio CORCE, 3er piso, Col. Doctores, Ciudad de México, 06725 México

**Keywords:** Insomnia, Rural population, Urban area, Health-related quality of life, Excessive sleepiness, Sleep quality

## Abstract

**Background:**

Sleep disorders are often associated with reduced physical and mental well-being in older adults. Although regional variations in sleep disorders have been observed, evidence remains limited as to how these differences may relate to health-related quality of life (HRQoL), especially in rural populations. The objective was to determine whether the association between insomnia, poor sleep quality, sleepiness and naps with HRQOL is modified according to place of residence (rural vs. urban).

**Methods:**

Cross-sectional data from two cohort studies in Mexico were used: the Cohort of Rural Frailty (2018; *n* = 514) and the Cohort of Obesity, Sarcopenia and Frailty of Older Mexican Adults (2019; *n* = 431). Sleep disorders and related factors were measured through the Athens Insomnia Scale, Pittsburgh Sleep Quality Index, Epworth Sleepiness Scale, and self-reported long naps (≥ 1 h per episode). HRQoL was assessed with the SF-36 Health Survey. Linear regression models were fitted for the proposed associations.

**Results:**

In rural areas, insomnia and poor sleep quality were significantly associated with lower HRQoL scores in both physical and mental components; while long naps (≥ 1 h per episode) were associated with lower scores on the physical component, and sleepiness with the mental component. No significant relationships were observed for urban area residents.

**Conclusions:**

Insomnia, long naps (≥ 1 h per episode), and poor sleep quality reduce HRQoL of older adults in rural areas, these findings have important implications for public health policies, that should focus in improving awareness, detection and treatment of sleep disorders by primary care especially in vulnerabilized areas such as rural Mexico.

## Background

Sleep disorders have an impact on physical and mental health, especially in the older people, among whom insomnia is the most common sleep disorder, affecting 30–50% with insomnia symptoms and 12–20% with a clinical diagnosis [1]. Several studies have reported negative associations between extremes of sleep duration or poor sleep quality and health-related quality of life (HRQoL) in older adults [2–4]. However, this association is still of interest due to the aging process being heterogeneous; it has been reported that there is variability in sleep and its disorders depending on the place of residence [5–7].

A study analyzing national data on Mexican adults over the age of 20 found that residents of urban areas reported a higher prevalence of insomnia and an increased risk of obstructive sleep apnea syndrome compared to those living in rural areas [5]. Another study focused on American people aged over 50 years old documented that, in rural areas, older adults tend to sleep more than their urban counterparts [6]. Likewise, a meta-analysis conducted in an older adult population in China reported that individuals in rural areas had a higher prevalence of sleep disorders than those in urban areas [7]. On the other hand, research in older adults over 60 years old in China found that poor sleep quality and daytime sleepiness were negatively associated with HRQoL regardless of residential region (urban versus rural) [8].

Despite previous studies, the evidence on the association between sleep and HRQoL in older adults that considers place of residence is insufficient and has focused mainly on sleep duration, without addressing other disorders such as insomnia, daytime sleepiness, or naps. In the particular case of Mexico, with regard to the differences between rural and urban areas, a recent study observed that rural older adults exhibit lower educational attainment, greater ethnic diversity, greater functional health deterioration, and less access to health care than their urban counterparts [9]. These results are consistent with another study of older adults in Mexico that analyzed longitudinally health inequalities and showed that women, the poorest, and residents of rural areas consistently had worse health conditions such as depression, sarcopenia, frailty, dependence in basic and instrumental activities of daily living, and falls [10]. This situation can be explained by the accumulation of gaps in education, social security, housing, nutrition, and income that characterize rural areas of the country. Therefore, it is possible to hypothesize that the presence of sleep problems in rural older adults in Mexico has a greater impact on their quality of life.

Therefore, the aim of this study was to determine whether the association between sleep disorders (insomnia, poor sleep quality, sleepiness, and napping) and health-related quality of life is modified according to place of residence (rural vs. urban) in community-dwelling Mexican older adults.

## Methods

This study conducted a secondary data analysis using information from two cohort studies on aging and health in Mexico: The Cohort of Rural Frailty (CRF) and the Cohort of Obesity, Sarcopenia and Frailty in Older Mexican Adults (COSFOMA). The CRF began in 2009 with 600 individuals aged ≥ 65 years living in rural localities (< 2500 inhabitants) in Mexico [11] and has three waves (2009, 2013 and 2018) with sociodemographic information, health and geriatric assessments. In the 2018 wave, a specific module was included with sleep topics: insomnia, sleep quality, sleepiness and naps. COSFOMA recruited 1252 people ≥ 60 years old entitled to the Mexican Social Security Institute (IMSS by its Spanish acronym) in Mexico City in 2014. COSFOMA has collected sociodemographic, health, and geriatric assessment data annually from 2014 to 2019. In 2019, the same sleep-related module that had been evaluated in the 2018 CRF study was incorporated in COSFOMA [12].

This study analyzes cross-sectional data from both studies (CRF-2018 and COSFOMA-2019). To ensure comparability between study samples, only participants aged 70 and over with complete data on key variables were included, because CRF-2018 does not include participants aged between 60 and 69 years. In total, the final sample comprised 514 participants from CRF and 431 from COSFOMA.

Participants in both studies were informed of the research procedures, and they signed a letter of informed consent prior to participation. The National Institute of Public Health Research and Ethics Committees approved CRF (Number 1159). The IMSS National Scientific Research Committee approved COSFOMA (R-2018-785-023).

### Health-related quality of life

Health-related quality of life (HRQoL) was measured using the questionnaire SF-36. This questionnaire consists of 36 items measuring eight dimensions of HRQoL: physical function, physical role, bodily pain, general health, vitality, social function, emotional role and mental health. Out of the first four, a score is generated that includes the quality of life of the physical component; and out of the remaining four, the mental component. The values of these components range from 0 to 100, where higher scores indicate better quality of life [13]. Both components were considered as dependent variables. These summary components offer a psychometric simplification of the health profile that accounts for over 80% of the reliable variance found within the eight dimensions of HRQoL [14]. The SF-36 has been validated in Mexico exhibiting satisfactory reliability with Cronbach’s alpha values ranging from 0.79 to 0.87 [15]. Some discrepancies have been identified in factor analyses studies with four [16] or six-factor structures [15].

### Sleep variables

#### Insomnia

The Athens Insomnia Scale (AIS) was used, which consists of eight items, each of which can have a score ranging from 0 to 3, where 0 corresponds to no problem and 3 to a very serious problem (did not sleep at all). The total score ranges from 0 to 24, where higher values imply greater insomnia problems. Insomnia was defined as an AIS score of ≥ 6 [17]. The AIS has demonstrated strong internal consistency. The original and the Mexican validation of the AIS reported Cronbach’s alpha values around 0.90, with mean item–total correlation coefficients of approximately 0.70 [17, 18]. Factor analyses have consistently indicated a unidimensional structure [17, 18].

#### Sleep quality

The Pittsburgh Sleep Quality Index (PSQI) was included. The PSQI is comprised of 18 questions that assess the presence of seven components of sleep quality: (1) subjective sleep quality, (2) sleep latency, (3) sleep duration, (4) habitual sleep efficiency, (5) sleep disturbances, (6) use of sleep medications, and (7) daytime dysfunction. Each component of the PSQI is scored on a scale from 0 to 3, yielding a total score ranging from 0 to 21. A score > 5 indicates poor sleep quality [19]. A systematic review and meta‑analysis confirmed that the PSQI has good reliability (Cronbach’s alpha value around 0.80) and validity across both clinical and non‑clinical samples, although some discrepancies have been identified in factor analyses studies [20]. The Mexican PSQI validation study showed satisfactory reliability (Cronbach’s alpha = 0.78) and significant correlations (*r* = 0.53–0.77) between component scores and the PSQI’s global index, supporting a two‑factor structure [19].

#### Sleepiness

The Epworth Sleepiness Scale (ESS) was used to evaluate daytime sleepiness. The ESS is an eight-question questionnaire that measures the propensity to fall asleep in everyday daytime situations. Each question is answered on a scale of 0–3, where 0 means no probability of falling asleep and 3 means high probability. Overall score ranges from 0 to 24. Excessive sleepiness was determined if the score was greater than 12 [21]. The ESS original validation study reported strong internal consistency (Cronbach’s alpha = 0.88) and strong discriminative validity, distinguishing healthy individuals from patients with narcolepsy or obstructive sleep apnea [22]. Subsequent work confirmed its stability, with test-retest reliability of *r* = 0.82 over a five‑month interval [21]. In Mexico, the ESS has likewise demonstrated strong reliability (Cronbach’s alpha = 0.89) and a single‑factor structure, with scores differentiating narcoleptic and sleep apnea patients from good sleepers [23].

#### Naps

The episodes of long naps were determined if participants reported taking naps lasting ≥ 1 h regularly per episode [24].

### Covariates

We include sociodemographic and health covariates that are associated with HRQoL [2–4, 25]. Both studies included: age (70–79 vs. 80+), sex, marital status (with partner vs. without partner) and current employment status. Multimorbidity was defined through self-reporting of two or more chronic diseases diagnosed by a physician (based on the specific conditions of each cohort). The presence of depressive symptoms was assessed with the 15-item version of the Geriatric Depression Scale (GDS) in the CRF [26], and the presence of depressive symptoms was considered if GDS ≥ 6. In COSFOMA, the 35-item version of the Center for Epidemiologic Studies Depression Scale-Revised (CESD-R) was applied with a cut-off point equal to 57 points for significant depressive symptoms [27]. In both studies, polypharmacy was considered if participants referred the consumption of ≥ 3 medications. The presence of difficulty in performing basic activities of daily living (BADL) was defined if the participant reported requiring support in at least one of the six basic personal activities of daily living (bathing, dressing, toileting, mobility, continence and feeding) [28]. In the CRF study, physical activity was classified into three levels based on the International Physical Activity Questionnaire (IPAQ): low, moderate and vigorous [29]. To ensure comparability between the two studies, low physical activity in CRF was defined as the “low” activity level. In COSFOMA, physical activity was assessed using the Physical Activity Scale for the Elderly (PASE) [30], with low physical activity defined as being in the sex-adjusted lowest quartile of the PASE distribution.

### Statistical analysis

Descriptive statistics were used using median with 25th-75th percentile and percentages when appropriate. Chi-square and Kruskal-Wallis tests were used to compare CRF and COSFOMA participants. In order to analyze the two components of HRQoL according to sleep variables (insomnia, poor sleep quality, sleepiness and naps), Kruskal-Wallis tests were performed. Linear regression models were employed to analyze the association between sleep variables and the physical and mental component of HRQoL, using separate models for each component. To explore possible differences by type of residence, we included the interaction between each of the sleep variables and a dichotomous indicator variable representing place of residence (1 = CRF (rural), 0 = COSFOMA (urban)). A covariate-adjusted model was specified for each HRQoL component. Multicollinearity was assessed using variance inflation factors (VIF) considering values of 10 indicating a problem. For both the physical and mental models, the highest VIF was 4.93, and the mean VIF was 2.1. Therefore, there was no evidence of multicollinearity. The goodness of fit was evaluated using adjusted R². Residuals were examined for normality, homoscedasticity, and independence using graphical methods (residual plots, Q-Q plots) and statistical tests (Shapiro-Wilk, Breusch-Pagan, and the White test). In the physical component model, the Shapiro–Wilk test showed no evidence of significant deviation from normality in the residuals (W = 0.998, *p* = 0.175). However, the Breusch–Pagan test indicated heteroscedasticity (χ² (1) = 3.84, *p* = 0.0499), and the White test confirmed its presence (χ²(146) = 283.12, *p* < 0.001). In the mental component model, the Shapiro–Wilk test showed a significant deviation from normality (W = 0.976, *p* < 0.001). Although the Breusch–Pagan test did not detect heteroscedasticity (χ² (1) = 0.06, *p* = 0.8097), the White test showed heteroscedasticity (χ²(146) = 216.28, *p* < 0.001). Therefore, both models were re-estimated using robust standard errors. Statistical analyses were performed using Stata 17.0 software (StataCorp LP, Texas, USA). Differences were considered statistically significant if the value of *p* < 0.05.

## Results

The characteristics of the participants in both studies are shown in Table 1. The CRF study sample consisted of a higher proportion of women (55.1%) and octogenarians (63.2%) than COSFOMA (45.5% and 23.2%, respectively). It was found that 65.9% of the participants reported that they were currently working in COSFOMA while in CRF it was 9.5%. Difficulty in performing BADLs and low physical activity were more present in rural people compared to urban; while polypharmacy was more present in urban compared to rural participants. Regarding sleep disorders, insomnia was more frequent in COSFOMA than in CRF (30.2% vs. 24.1%, *p* = 0.04). Poor sleep quality was higher in CRF than in COSFOMA (68.7% vs. 49.4%, *p* < 0.01). No significant differences were observed in daytime sleepiness (*p* = 0.15) or in the frequency of long naps (≥ 1 h per episode) (*p* = 0.86).

**Table 1:** Characteristics of the study participants of CRF-2018 and COSFOMA-2019, Mexico

	CRF-2018		COSFOMA-2019		
	*n* = 514		*n* = 431		
Variables	*n*	%	*n*	%	*p*-value*
Women, %	283	55.1	196	45.5	0.003
Age group, %					
70–79 years	187	36.4	331	76.8	< 0.001
80 + years	327	63.2	100	23.2	
With partner, %	211	41.1	237	55.0	< 0.001
Currently working, (%)	49	9.5	284	65.9	< 0.001
Multimorbidity, %	195	37.9	149	34.6	0.284
Depressive symptoms, %	178	34.6	36	8.4	< 0.001
Polypharmacy (≥ 3 medications), %	108	21.0	283	65.7	< 0.001
BADL limitations, %	141	27.4	73	16.9	< 0.001
Low physical activity, %	257	50.0	144	33.4	< 0.001
Independent variables					
Insomnia, %	124	24.1	130	30.2	0.037
Poor sleep quality, %	353	68.7	213	49.4	< 0.001
Daytime sleepiness, %	75	14.6	78	18.1	0.145
Long naps (≥ 1 h per episode), %	41	8.0	33	7.7	0.855
Outcome variables	Median	**(P25**,** P75)**	Median	**(P25**,** P75)**	
Health-related quality of life (SF-36)					
Physical component summary (0-100)	53.6	(30.7, 76.2)	50.3	(41.3, 54.5)	< 0.001
Mental component summary (0-100)	78.4	(59.3, 90.0)	53.7	(48.1, 58.6)	< 0.001

*CRF* Cohort of Rural Frailty, *COSFOMA* Cohort of Obesity, Sarcopenia and Frailty of Older Mexican Adults, *BADL,* basic activities of daily living

P25: 25^th^ percentile

P75: 75^th^ percentile

*Chi-square or Kruskal-Wallis test

Overall, older adults with insomnia, poor sleep quality, daytime sleepiness and those who reported taking long naps (≥ 1 h per episode) exhibited lower scores on both components of HRQoL compared to the group that did not present any of the sleep disorders, and this was especially clearer among individuals residing in rural areas (Table 2).

**Table 2:** Physical and mental health component scores of health-related quality of life by sleep characteristics

Independent variables	CRF-2018						COSFOMA-2019						
	Physical component			Mental component			Physical component				Mental component		
	Median	(P25, P75)	*p*-value*	Median	(P25, P75)	*p*-value*	Median	(P25, P75)	*p*-value*		Median	(P25, P75)	*p*-value*
Insomnia													
Yes	33.3	(18.8, 53.9)		59.6	(39.6, 74.5)		43.9	(35.1, 52.3)		50.5		(43.4, 56.3)	
No	59.5	(38.6, 77.6)	< 0.001	82.9	(68.6, 91.4)	< 0.001	51.3	(46.0, 55.1)	< 0.001	54.5		(49.9, 59.0)	< 0.001
Poor sleep quality													
Yes	47.1	(25.0, 72.1)		71.4	(53.2, 86.8)		47.4	(35.7, 52.6)		53.2		(47.0, 57.9)	
No	67.6	(47.6, 79.8)	< 0.001	86.8	(77.1, 95.7)	< 0.001	52.4	(46.7, 55.6)	< 0.001	54.2		(49.9, 58.8)	0.053
Daytime sleepiness													
Yes	46.9	(22.4, 62.4)		58.6	(40.0, 77.1)		45.3	(35.9, 52.3)		52.8		(46.1, 57.2)	
No	54.8	(33.3, 76.7)	< 0.001	80.7	(62.9, 91.4)	< 0.001	50.8	(43.0, 54.9)	< 0.001	53.9		(48.7, 58.8)	0.122
Long naps (≥ 1 h per episode)													
Yes	38.6	(25.2, 48.6)		67.1	(44.6, 82.9)		51.5	(46.0, 52.8)		54.4		(46.8, 57.4)	
No	54.8	(32.4, 76.4)	< 0.001	78.6	(60.7, 91.4)	0.003	50.2	(40.1, 54.8)	0.805	53.6		(48.4, 58.6)	0.444

*CRF* Cohort of Rural Frailty, *COSFOMA* Cohort of Obesity, Sarcopenia and Frailty of Older Mexican Adults

P25: 25^th^ percentile

P75: 75^th^ percentile

*Kruskal-Wallis test

Table 3 presents the results of the linear regression models including the interaction term between the sleep variables and place of residence. For both models, at least one of the interaction terms was statistically significant, showing that place of residence modified the association between the variables of interest. As for the rural area, the presence of insomnia (β = -8.6; 95% CI: -12.6,-4.7), poor sleep quality (β = -4.0; 95% CI: -7.5, -0.5), and taking long naps (β = -9.3; 95% CI:-14.8, -3.7) were associated with lower scores on the physical component of quality of life; while the presence insomnia (β = -10.9; 95% CI:-14.6,-7.2), poor sleep quality (β = -4.9; 95% CI:-7.6,-2.2) and sleepiness (β = -4.7; 95% CI:-9.1,-0.4), in rural people, was associated with lower scores in the mental component of the quality of life. On the other hand, no significant associations were observed for any of the sleep variables in the case of urban area residents.

**Table 3:** Modification of the association between sleep variables and physical and mental components of HRQoL in two Mexican cohorts of older adults

	Model 1*			Model 2*		
	Outcome: Physical component of HRQoL			Outcome: Mental component of HRQoL		
	β	(95% CI)	*p*-value^‡^	β	(95% CI)	**p-value** ^**‡**^
Insomniaª			< 0.001			<0.001
CRF study (rural)	-8.6	(-12.6, -4.7)		-10.9	(-14.6, -7.2)	
COSFOMA study (urban)	0.3	(-2.4, 2.9)		-0.7	(-3.1, 1.7)	
Poor sleep quality^b^			0.027			<0.001
CRF study (rural)	-4.0	(-7.5, -0.5)		-4.9	(-7.6, -2.2)	
COSFOMA study (urban)	-2.1	(-4.4, 0.1)		0.3	(-1.6, 2.3)	
Sleepiness^c^			0.585			0.032
CRF study (rural)	1.2	(-3.2, 5.6)		-4.7	(-9.1, -0.4)	
COSFOMA study (urban)	0.03	(-2.9, 3.0)		0.5	(-2.1, 3.0)	
Long naps (≥ 1 h per episode)^d^			0.003			0.163
CRF study (rural)	-9.3	(-14.8, -3.7)		-4.1	(-9.9, 1.7)	
COSFOMA study (urban)	1.5	(-2.1, 5.0)		-2.6	(-5.9, 0.8)	
Adjusted R²	0.473			0.610		

*HRQoL* Health-Related Quality of Life, *CRF* Cohort of Rural Frailty, *COSFOMA* Cohort of Obesity, Sarcopenia and Frailty of Older Mexican Adults, *CI* Confidence Interval

ª Also adjusted for poor sleep quality, daytime sleepiness, and long naps

^b^ Also adjusted for insomnia, daytime sleepiness, and long naps

^c^ Also adjusted for insomnia, poor sleep quality, and long naps

^d^ Also adjusted for insomnia, poor sleep quality, and daytime sleepiness

*Multiple linear regression model adjusted for sex, age, marital status, currently working, multimorbidity, depressive symptoms, polypharmacy, BADL limitations, and low physical activity

^‡^ p-Value for interaction

The goodness of fit of the regression models was acceptable. The adjusted R² for the physical HRQoL component was 0.473, indicating that 47.3% of the variance was explained by the variables. For the mental HRQoL component, the adjusted R² was 0.61 meaning that 61.0% of the variance was accounted for. The overall F-tests were statistically significant (*p* < 0.001) in both cases.

## Discussion

The findings of this study suggest that place of residence (rural vs. urban) plays a significant role in the association of sleep disorders and HRQoL in older adults.

Among rural residents, insomnia and poor sleep quality were associated with lower HRQoL in both the physical and mental components. Additionally, taking long naps (≥ 1 h per episode) was associated with lower physical HRQoL, while daytime sleepiness was associated with lower scores in the mental component.

This study revealed a significant independent association between insomnia and reduced HRQoL, even after adjusting for sociodemographic and health variables, as well as other sleep-related disorders such as sleep quality, prolonged napping, and daytime sleepiness. Previous literature has reported an association between insomnia and poor HRQoL, the studies have been conducted mainly in adults over 18 years old in Sao Paulo, Canada or France [25, 31, 32]. Fewer studies were found in older people, but those that do exist have reported that insomnia is associated with lower HRQoL. Some observed this relationship across all HRQoL domains in data on Americans or Greeks [33, 34], while others focused on total scores in Norway older people [35]. Additionally, one study found that the interaction between insomnia and depressive symptoms was associated with reduced HRQoL [36].

The presence of insomnia in rural settings was associated with reductions in both physical and mental components of HRQoL, exceeding the threshold for minimum clinically important difference (5 points). Importantly, insomnia is a treatable condition in older adults; interventions such as cognitive behavioral therapy in weekly 2-hour sessions for 4 months have been shown to reduce biological markers of cardiovascular risk at both the end of the intervention and one-year follow-up [37].

Our work finds that low sleep quality is related to worse quality of life in the physical and mental component for rural older adults. Our results are consistent with those reported by a recent systematic review and meta-analysis in which they observed that self-reported good sleep quality is linked to older adults’ perception of their quality of life [38]. One of the problems with poor sleep quality is that it can be an indicator of significant sleep fragmentation. On the other hand, fragmentation contributes to excessive daytime sleepiness, affecting daily life by making people less able to do their activities during the day [38].

The long naps (≥ 1 h per episode) was associated with and lower scores on the physical component of HRQoL, again in rural areas. Napping has been the subject of much debate. On the one hand, it is suggested that naps may benefit cognitive function and well-being [39, 40]. On the other hand, it has been documented that long naps are associated with increased risk of hypertension and diabetes [40]. Long naps, especially in those with normal nighttime sleep time, may reflect sleep fragmentation and underlying health problems, so naps may have different effects depending on their duration and overall sleep quality. Additionally, long naps, especially after noon, decrease the need for sleep at night, leading to difficulty falling asleep and a perverse pattern of sleep health. Short, restorative naps may be beneficial [39], whereas prolonged naps may be associated with health risks, including frailty and low HRQoL [41, 42].

Our findings support the hypothesis that the presence of sleep problems is linked to quality of life in rural older adults. The associations observed in the rural sample but not in the urban sample can be explained by the influence and the context of social and economic vulnerability faced by the older residents of these rural contexts that exacerbate the negative effects of sleep disorders on health-related quality of life [11, 43]. A systematic review and previous studies have shown that socioeconomic status (SES) correlates with sleep disorders. Individuals with low SES are more likely to engage in unhealthy behaviors, have lower educational attainment, experience higher levels of stress due to work or financial insecurity, and have limited access to healthcare programs and resources; these factors increase the likelihood of insomnia or other sleep disorders [7, 44]. In the case of Mexico, the health inequality observed in rural areas is partly the result of a health system with a long history of centralization and concentration of medical personnel in urban areas, which has maintained exclusion and increased incidence of preventable diseases in rural areas, which are often the most impoverished [45]. This could be correlated with the differences detected in this study. Research in other contexts has found similar results, such as poor sleep quality in rural areas of Iran [46], although generalization should be done with caution due to methodological and sociocultural differences.

Currently, there is a surge in the study of environmental settings and urban design that directly affects health [47]. In Latin America, cities are recognized as having a high prevalence of sleep problems related to noise, pollution, and daily stress [48]. It has been described that noise in cities promotes chronic stress, which negatively affects physiological parameters linked to sleep [49, 50]. These, in conjunction with our findings, reinforces the need to expand sleep and quality of life research, incorporating environmental and urban design elements, including healthcare access, socioeconomic, and cultural practices, additional sleep disorders such as sleep apnea, and using nationally representative designs to strengthen generalizability.

Our findings are consistent with Grandner et al.‘s socioecological model of sleep health [51, 52], according to which sleep is influenced by individual level factors (attitudes, psychology, health, etc.), but these are integrated into the context of social level factors (work, neighborhood, socioeconomic status, etc.), and the latter are also integrated into the context of societal level factors (environment, technology, geography, natural environment, etc.). In this model, sleep exists at the intersection of upward influences (individual, socially integrated, societal integrated) and downward influences that encompass the combination of health outcomes and functioning/performance [51]. Taking into account the contextual factor of place of residence and its implications for people’s health and quality of life, public policy design must be considered.

Public health policy addressed to rural areas in Mexico would include improving awareness of sleep disorders and promotion of sleep health in older adults in first contact health personal (nurses, physicians, health promoters), integrate sleep health questions as a part of routine evaluation in this population, awareness campaigns to educate the community about healthy sleep habits and behaviors, improving access to treatment of sleep disorders in rural areas through telemedicine where feasible; hopefully in line with the socioecological model improve access to appropriate housing, safety and financial security in rural areas [53].

This study has several limitations. First, the cross-sectional design limits the ability to infer causal relationships, or even to rule out the possibility of reverse causality in the observed associations. The second limitation is that daytime sleep (naps) comes from self-reporting, so it cannot be ruled out that information on napping is subject to social desirability. The third limitation is that some of the study covariates, such as depression and physical activity, were not assessed with the same scales. Fourth, CRF and COSFOMA do not represent the rural and urban context, respectively, as each study was designed with distinct sampling strategies to fulfill their original aims, rather than to represent the complete diversity of rural and urban older adults in Mexico. Fifth, the comparison between two different cohorts introduces potential selection bias because of different sampling frames: COSFOMA captures IMSS-affiliated urban adults representing a population with formal insurance, a history of employment in the formal sector, and access to healthcare, whereas CRF targets rural residents with low socioeconomic resources and inadequate health service infrastructure. Sixth, housing characteristics were not measured, limiting the investigation of environmental pathways (such as ventilation and noise) that underline rural-urban differences. Seventh, the analyses are restricted to adults aged 70 and over, and therefore we cannot determine the behavior of younger adults (aged 60–69) in relation to sleep problems.

In conclusion, our findings underscore the importance of incorporating sleep-related assessment and treatment into primary health care for older adults, especially in cases of poor sleep quality, insomnia, or napping for one hour or more. Non-pharmacological strategies such as health education, cognitive behavioral therapy, and appropriately timed physical activity should be prioritized to mitigate the impact of sleep disturbances, with particular attention to insomnia.

## Data Availability

Data are available from the authors upon reasonable request.

## Abbreviations

HRQoL: Health-related quality of life
CRF: Cohort of Rural Frailty
COSFOMA: Cohort of Obesity, Sarcopenia and Frailty in Older Mexican Adults
AIS: Athens Insomnia Scale
PSQI: Pittsburgh Sleep Quality Index
ESE: Epworth Sleepiness Scale
GDS: Geriatric Depression Scale
CESD-R: Center for Epidemiologic Studies Depression Scale-Revised
BADL: Basic activities of daily living
IPAQ: International Physical Activity Questionnaire
PASE: Physical Activity Scale for the Elderly
